# 943 Predictors of Post-Traumatic Stress Disorder in Burn Injury Survivors: A Retrospective Analysis

**DOI:** 10.1093/jbcr/iraf019.474

**Published:** 2025-04-01

**Authors:** Anh-Tho Antoinette Nguyen, Keith Sweitzer, Derek Bell

**Affiliations:** Kessler Burn Center; Kessler Burn Center; Kessler Burn Center

## Abstract

**Introduction:**

Burn injuries are traumatic events that can lead to both substantial physical impairment and profound psychological consequences. Among these, Post-Traumatic Stress Disorder (PTSD) is a prevalent mental health outcome in burn injury survivors. This study aims to investigate the demographic, clinical, and injury-related factors that contribute to the development of PTSD in burn survivors, offering a deeper understanding of its psychological impact.

**Methods:**

This retrospective chart review analyzed data from 95 patients treated at an ABA-verified burn center between January 2017 and December 2020. All patients had documented mental health conditions either prior to or following their burn injury. Data collected included patient demographics, burn injury characteristics (total body surface area [TBSA] involved, degree, and anatomical location of burns), and comorbidities. PTSD diagnoses were determined based on DSM-5 criteria recorded in patient charts.

**Results:**

PTSD was diagnosed in 20% of burn survivors included in the study. Key predictors for PTSD included burns affecting more than 10% of TBSA (P < 0.05), burns involving the head or face (P < 0.05), and prolonged hospital stays (P < 0.05). A significant gender difference emerged, with female survivors showing a higher incidence of PTSD compared to males (P < 0.05). This finding aligns with broader literature, suggesting that females may have increased susceptibility to PTSD due to a combination of psychosocial and neurobiological factors, as well as differences in stress response. Pre-existing psychiatric conditions, particularly anxiety and depression, were also strongly associated with PTSD development (P < 0.05). Additionally, patients under the age of 35 at the time of injury were more likely to develop PTSD (P < 0.05). In contrast, the number of surgical interventions was not found to be a significant predictor of PTSD incidence, indicating that psychological outcomes are influenced by factors beyond the intensity of medical treatment.

**Conclusions:**

The results of this study underscore the significant psychological impact of burn injuries, with several key factors predicting PTSD development, including female gender, larger TBSA involvement, head or facial burns, extended hospital stays, and pre-existing psychiatric conditions. These findings highlight the importance of integrating routine psychological screening and early interventions into the standard care of burn survivors.

**Applicability of Research to Practice:**

The study emphasizes integrating psychological assessments into burn care, particularly for high-risk groups like females, younger patients, and those with extensive burns or pre-existing mental health conditions. Early PTSD identification allows for timely interventions like TF-CBT. Multidisciplinary care and trauma-informed education for healthcare providers can enhance overall recovery and mental health outcomes for burn survivors.

**Funding for the Study:**

N/A

